# The Appropriateness of Empirical Antimicrobial Treatment of Uncomplicated Urinary Tract Infection in Adult Female Patients in Jazan Region, Saudi Arabia

**DOI:** 10.3390/clinpract13040067

**Published:** 2023-07-03

**Authors:** Majid A. Darraj

**Affiliations:** The Department of Internal Medicine, The Faculty of Medicine, Jazan University, Jazan 45142, Saudi Arabia; mdarraj@jazanu.edu.sa

**Keywords:** urinary tract infection, antimicrobial susceptibility pattern, uropathogens, Jazan

## Abstract

Introduction: Uncomplicated urinary tract infection (uUTI) is one of the most prevalent bacterial infections in clinical practice. Empirical treatment is used commonly; thus, knowledge of the local antimicrobial susceptibility pattern is crucial to avoid the growing antibiotic resistance. Purpose: The aim of this study is to evaluate the effectiveness of the empirical treatment of uUTI and determine the susceptibility pattern for common uUTI causative microorganisms at outpatient clinics in hospitals in the Jazan region. Method: This is a prospective observational study of 120 patients with uncomplicated urinary tract infections attending internal medicine outpatient clinics in Al-Hayat Jazan hospital, Saudi Arabia, from September 2021 to January 2023. Results: In total, 120 patients were included in the study. The mean age of the participants was 48.53 ± 9.29. Risk factors for UTI were found in 57.5%, and type 2 diabetes mellitus (DM) was the major risk factor (27.5%). The most common causative microorganism was *Escherichia coli* (*E. coli*) (87.5%), followed by *Klebsiella pneumoniae* (5%), *Staphylococcus aureus*, *Enterobacter* spp., and *Enterococcus* spp. (2.5%). Nitrofurantoin was the most effective antimicrobial agent (sensitivity rate of 91.7%) against all microorganisms, followed by Ciprofloxacin (75%). Conclusions: This study concluded that nitrofurantoin and Ciprofloxacin are suitable empirical treatments for uncomplicated urinary tract infection in the Jazan region, and increased resistance against trimethoprim/sulphamethoxazole (TMPSMX) and cefaclor was observed; thus, empirical therapy with these agents should be reconsidered in local guidelines. Wide surveillance research is necessary to monitor effective empirical therapies and to evaluate the regional antimicrobial susceptibility pattern.

## 1. Introduction

Urinary tract infection (UTI) is one of the most prevalent bacterial infections in general practice and accounts for 1–6% of medical referrals [1]. Acute uncomplicated cystitis is the most common UTI. Frequency and dysuria without vaginal discharge [1,2,3] in an immune-competent female of reproductive age without other associated morbidities or urinary tract defects or abnormalities are characteristics of acute uncomplicated cystitis [3]. The clinical evaluation usually is unremarkable apart from suprapubic discomfort and tenderness, and in order to make the diagnosis, a urine analysis is recommended rather than a urine culture [1,3]. A urine culture is usually required for patients with clinical features suggesting acute pyelonephritis and those who did not respond to treatment or with a recurrent UTI after two to four weeks of optimum antibiotic therapy or for women with atypical manifestations [2,3].

Acute uncomplicated cystitis is among the main prevalent reasons for women in otherwise healthy communities to have an antibiotic prescription. Despite established criteria for the best antimicrobial drugs of choice and the duration of treatment, studies show that prescribing practices vary widely [3]. Although it is not a serious disease, acute uncomplicated cystitis has effects on a patient’s quality of life [2].

Since 1999, when the Infectious Diseases Society of America (IDSA) published a practice guideline on the management of females with acute uncomplicated cystitis and pyelonephritis, the resistance of organisms against antibiotics has risen, recognition of the role of antimicrobial therapy’s environmental side effects has grown, newer medications and management durations have been investigated, and clinical responses have become more widely reported. In addition, several studies included females with microorganisms that were resistant to the drugs for the assessment of the expected effectiveness ratio in clinical practice, where empirical treatment is provided without a urine culture or before these data are available [3,4].

The microorganisms responsible for causing uncomplicated cystitis mainly include *E. coli*, which represents about 30% to 90% of uropathogens [1,2], in addition to those such as *Klebsiella pneumoniae*, *Enterobacter*, *Proteus mirabilis*, *Pseudomonas*, *Enterococcus*, and *Staphylococcus saprophyticus* [1,5,6]. Uncommon pathogens include *Citrobacter species* [5].

However, some research has demonstrated that *E. coli* vulnerability profiles differ by clinical context, such as intensive care unit vs. inpatient ward vs. outpatient sites, at a given facility [7,8].

Antimicrobial treatment for a UTI should ideally give consistent activity against the bacteria found in the patient’s urine. To do so, medication should be chosen with consideration of the potential infections, microorganisms, and their antimicrobial susceptibility characteristics [6].

Although a urinary tract infection (UTI) is a common cause of antibiotic use, little is known about the appropriate duration of treatment. For asymptomatic bacteriuria and/or pyuria, a high-quality study suggested delaying the initiation of antibiotic agents for bacteriuria without symptoms and recommended a short-duration treatment for an uncomplicated urinary tract infection (uUTI) [9].

As reported by the 2010 IDSA updated guideline, nitrofurantoin, 100 mg, two times daily for five days; TMPSMX, 160/800 mg, twice per day for three days; or fosfomycin, 3 g of a single dose are considered the drugs of choice for the treatment of an uncomplicated urinary tract infection [1]. The selection of these drugs must be customized and depends on the patient’s hypersensitivity and adherence, the local practice, the frequency of susceptibility and drug resistance in the local population, availability, and price [5,9].

Flouroquinolones, amoxicillin/clavulinic acid, and cefaclor are recommended as alternative second-line therapies if first-line drugs cannot be administered or if the patient is intolerant [5,7].

This is why understanding the causative microorganisms of UTIs and their patterns of antimicrobial resistance in particular geographic areas may help in selecting the proper empirical antibiotic treatment. Since there has not been much research on the antimicrobial susceptibility pattern of UTIs among females in Jazan in recent years, the current study was carried out to ascertain the local antimicrobial susceptibility patterns of frequently prescribed antibiotics among females in the Jazan region of Saudi Arabia.

This study aims to evaluate the effectiveness of the empirical treatment of uUTIs and the susceptibility pattern for common uUTI causative microorganisms in the Jazan region of Saudi Arabia. Despite the readily apparent advantages of antimicrobial therapy for patients, misuse and overuse have led to an increase in uropathogenic bacterial resistance, posing a major concern for public health.

## 2. Methods

### 2.1. Study Design and Study Area

This was a prospective observational study carried out in internal medicine outpatient clinics involving 120 female patients with uUTIs who presented to Al-Hayat Jazan hospital from September 2021 to January 2023. 

### 2.2. Study Population and Sample Size

All patients with symptoms of urinary tract infection and diagnosed with a uUTI visiting the internal medicine outpatient clinics at Al-Hayat Jazan hospital during the study period from September 2021 to January 2023 were included in the study. 

Inclusion criteria: We included patients over 18 years old with uUTIs who were willing to participate and who signed the informed consent. Exclusion criteria: We excluded patients under 18 years old and women who were pregnant or on corticosteroids. Females who declined to provide the required information or who refused to give their consent were also excluded from the study.

### 2.3. Operational Definition

An uncomplicated urinary tract infection (uUTI) is defined as a UTI that is not associated with urinary tract structural abnormalities in non-pregnant females with the presence of symptoms [3].

The phrase “empiric therapy” describes the use of antibiotics before receiving the findings of a blood culture and an antibiotic susceptibility test [10]. Antimicrobial resistance (AMR) is a condition in which bacteria, viruses, fungi, and parasites evolve over time and cease to respond to antibiotics, making infections more difficult to cure and raising the risk of disease transmission, life-threatening sickness, and death [11].

A clinical cure is defined as the elimination of clinical symptoms under 4 white blood cells (WBCs) per high-power field on microscopy.

#### 2.3.1. Procedure

uUTI was assessed based on the clinical symptoms and laboratory assessment.

Standard techniques were used for the urine culture, and antimicrobial sensitivity was determined and interpreted in the microbiology laboratory. The participants were informed to wash their hands and genital area thoroughly and then to collect urine midstream in a sterile container; the samples were plated within 2 h of collection to reduce false-positive results and contamination.

#### 2.3.2. Cultures and Antimicrobial Susceptibility Test

Utilizing semiquantitative techniques, urine samples were inoculated on suitable culture media and then cultured for 48 h at 37 °C in an aerobic environment. The growth of the cultures was next assessed, and the number of colonies determined if the bacteriuria was substantial or not. Significant bacteriuria was defined as a growth of 10⁵ colony-forming units/mL, which is suggestive of a UTI [12]. According to CLSI Standards, antimicrobial susceptibility testing was carried out using the Modified Kirby Bauer Disc Diffusion technique on Muller Hinton Agar [13].

Before taking samples for culture, the patients were asked if they had taken any antibiotics prescriptions within the last five days. The empiric antimicrobial treatment was assessed for sensitivity according to the susceptibility testing. If the microorganisms were found to be susceptible to the antimicrobial treatment, treatment with it was deemed appropriate.

The empirical antibiotic was prescribed for the participants after urine samples for culture and sensitivity were taken and before the results of the culture were obtained. Four types of antibiotics according to the local practice, which included nitrofurantoin (Uvamin Retard, 100 Mg), Ciprofloxacin (CIFLOX 500 Mg), TMP SMX (Bactrim (sulfamethoxazole/trimethoprim) 800/160 mg), and cefaclor (Ceclor 500 mg), were used in the empirical treatment, and the sensitivity pattern was determined by using the MicroScan WalkAway 96 SI (Siemens, Munich, Bayern, Germany), according to the Clinical and Laboratory Standards Institute (CLSI) Document M100-S22.

### 2.4. Data Collection Tool

The patients were interviewed by a specialized nurse to collect the data. The data collection Excel sheet included background characteristics, such as age, sex, marital status, and risk factors, including hypertension, diabetes mellitus, renal stone, and urinary catheter. The microbiology laboratory results included a urine culture and antimicrobial sensitivity.

### 2.5. Data Management and Statistical Analysis

Data entry and analysis were performed using the Statistical Package for Social Sciences version 24 for Windows. The descriptive statistics were calculated in the form of frequency counts and percentages. The Chi square test was used to test the statistical significance of associations. A *p*-value of <0.05 was considered statistically significant.

### 2.6. Ethical Considerations

The study protocol was approved by the standing committee for scientific research at Jazan University (HAPO-10-Z-001). At the start of the study, the participants were informed about the objectives and procedures, side effects of the drugs, risks and benefits to participation, the length of the study, and the study supervisors’ contact information for the study through written informed (Arabic and English) consent. The participants were also informed that they could withdraw from the study at any time without any consequences or impact on their treatment and medical follow-up.

## 3. Results

In total, 133 patients with uUTIs were invited to participate in the study; 120 patients were enrolled in the study, as 13 were excluded either because they were pregnant (9 patients) or did not give consent (4 patients). The mean age of the participants was 48.53 ± 9.29. Risk factors for urinary tract infection were found in 57.5%. DM was the most common risk factor (27.5%), followed by renal stone (15%), hypertension (10%), and urinary catheter (5%). In 21.6% of cases, there was more than one risk factor. The background characteristics and risk factors are illustrated in Table 1.

**Figure 1:**
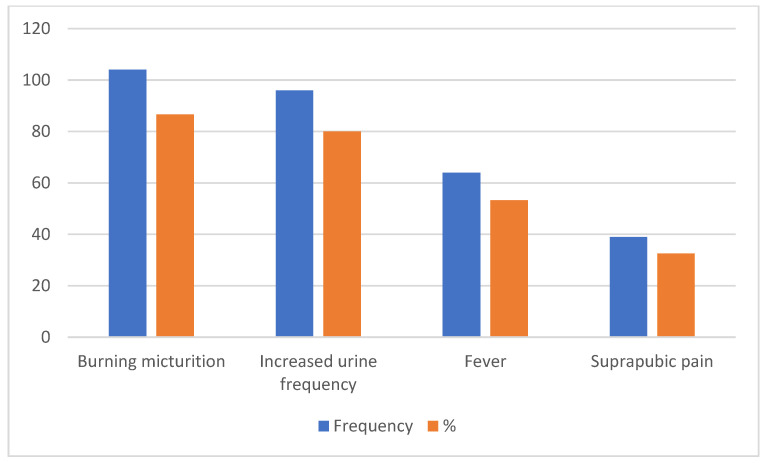
Presenting complaints for patients with uUTI.

A total of 86.7% of the patients presented with burning micturition, while an increase in urine frequency and lumbar/loin suprapubic pain and fever were the presenting complaints in 80%, 32.5%, and 53.3%, respectively (Figure 1).

**Figure 2:**
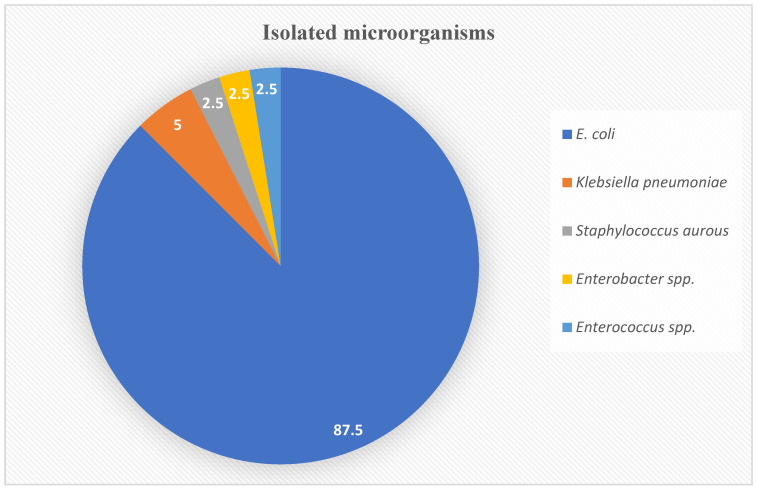
Isolated microorganisms percentages based on the urinary cultures.

The majority of the isolates were Gram-negative pathogens and accounted for 92.5% of all isolates. The most common causative microorganism isolated was *E. coli* (87.5%), followed by *Klebsiella pneumoniae* (5%) and *Staphylococcus aurous*, *Enterobacter* spp., and *Enterococcus* spp. at 2.5% each. The distribution of isolated uropathogens is shown in Figure 2.

Nitrofurantoin was the most effective antimicrobial agent (sensitivity rate of 91.7%), followed by fluoroquinolone (75%), TMPSMX (70%), and cefaclor (62.5%) (Table 2).

Antibiotic susceptibility patterns in relation to the isolated urinary tract pathogens are shown in Table 3. Nitrofurantoin showed a 92.4% sensitivity rate against *E. coli*, 83.3% sensitivity against *Klebsiella pneumoniae*, 66.7% sensitivity against *Enterobacter* spp., and 100% sensitivity against *Staphylococcus aurous*, and *Enterococcus* spp. Ciprofloxacin was significantly sensitive against *E. coli* (71.4%) and showed 100% sensitivity against *Klebsiella pneumoniae*, *Staphylococcus aurous*, *Enterobacter* spp., and *Enterococcus* spp. TMPSMX showed 68.6% sensitivity against *E. coli*, 50% sensitivity against *Klebsiella pneumoniae*, and 100% sensitivity against *Staphylococcus aurous* and *Enterococcus* spp. There was a significant sensitivity of Cefaclor (68.6%) against *Enterococcus* spp., while it was significantly resistant (100% sensitivity) against *Klebsiella pneumoniae*, *Staphylococcus aurous*, and *Enterobacter* spp. The correlation between antimicrobials and microorganisms using linear regression was illustrated in Table 4.

## 4. Discussion

In clinical settings, urinary tract infections are among the most common infections. These infections affect people of all age groups and sexes. Females are more prone to infection according to the demographic information. A short urethra makes women more susceptible to develop UTIs [8]. Some factors, such as pregnancy, elderly age, DM, surgery, immunosuppression, neurogenic bladder, nephrolithiasis, and structural abnormalities of the urinary tract, may raise the probability of developing a UTI [3,14,15]. An acute uncomplicated UTI is still considered one of the prevalent reasons for antibiotic prescription [3,15,16]. Although there are established guidelines for the best antimicrobial drug to be used, as well as the duration of treatment, studies have found that treatment practices vary widely [3,15]. The most common pathogen that causes a community-acquired UTI is *E. coli*, which accounts for about 95% [1,17,18], followed by *Proteus mirabilis*, *Kellebesia pneumoniae*, and *Staphylococcus saprophyticus* [18].

The current study found that *E. coli* was the most causative urinary organism (87.5%), followed by *Klebsiella pneumoniae* (5%) and *Staphylococcus aurous* (2.5%). This finding was consistent with a study from Riyadh by Balkhi et al. that showed the most common isolated organism was *E. coli* (52%), while *Klebsiella pneumonia* and *Pseudomonas aeruginosa* accounted for 15% and 8%, respectively [19].

Other research from hospitals in the USA and Europe demonstrated that the most prevalent microorganisms isolated were *E. coli* with 63.3% and 71.3%, *Klebsiella* spp. at 16.7% and 11.2%, and *Proteus mirabilis* at 6.4% and 5.0% in the USA and EU, respectively, as reported by Sader et al. [20].

The most common isolated pathogens as causative agents of UTIs in Saudi Arabia are *E. coli*, *Klebsiella pneumoniae*, and *Pseudomonas aeruginosa* [21,22]; *E. coli* was the most common microbe, followed by *Klebsiella pneumoniae*, isolated as uropathogens from the Gulf Corporation Council countries (GCC) area [23].

Thus, the findings from the current study were in accordance with the epidemiological data reported in the literature and from local, regional, and worldwide studies [21,22,23].

Generally, the frequency of UTIs is higher in young women and in the elderly in both sexes, as well as in association with instrumentation, such as a urinary catheter, and immunocompromised patients [22]. Females are more prone to develop UTIs [24,25], as mentioned above; hence, the aim of the study was to evaluate the effectiveness of empirical UTI antimicrobial therapy in women. Risk factors were found in 57.5%, with DM as the most common risk factor (27.5%), and this was supported by one study, which reported an overall 25.3% prevalence of UTIs in diabetes patients in Saudi Arabia [26].

Urinary tract infection occurs more frequently in type 2 DM [27,28] and is associated with severe disease and poor prognosis. UTIs are also more likely to be caused by microorganisms that are resistant to antibiotics [27]. Several immune system abnormalities, poor diabetic control, and autonomic bladder may all play a role in the increased risk of urinary tract infections [27,29].

Resistance to antibiotics against UTI-causative organisms such as Gram-negative microorganisms is growing; thus, the identification of the antibiotic-resistance characteristics of the pathogenic organisms in certain geographical regions is essential to consider when selecting a suitable empiric antibiotic treatment regimen [3,30,31].

Nitrofurantoin, fosfomycin, and mecillinam had the minimum recorded rates of resistance in lower UTIs, as per numerous research [1,18,32]. Nitrofurantoin as empiric therapy was found to have a higher sensitivity rate of 91.7% in the present study, which was consistent with a local report in KSA [33,34] and global guidelines and research [1,3,22,23,35,36].

Antibiotic insensitivity is widespread among bacteria that cause community-acquired UTIs. Specifically, TMPSMX resistance is rising [32]; thus, its use is based on the regional susceptibility pattern for *E. coli* and is only recommended when resistance is less than 20% [37]. This study showed that TMPSMX had 30% resistance. The antibiotics’ susceptibility pattern to TMPSMX reported in our current study was consistent with earlier investigations conducted in several Saudi Arabian districts [38,39,40] Observations from other studies in the USA and the United Kingdom also showed increased resistance to TMPSMX [35,41].

In contrast to several studies, in this current study, fluoroquinolone (ciprofloxacin) showed a significant cure rate in our region, with 75% sensitivity. Fluoroquinolone insensitivity was most widespread in Asia, especially China and Korea, where data from various studies showed a significant rise in ciprofloxacin resistance between 2008 and 2014 [30,42,43]. Fluoroquinolone is significantly effective in the treatment of uncomplicated UTIs; however, due to its tendency for collateral damage, it is recommended as an alternative drug when there is no other option [1,2,3]. Grigoryan et al. reported that fluoroquinolones were efficient therapy for UTIs, but they should be preserved for complicated infections to avoid bacterial resistance [44].

Cefaclor showed suboptimal results with 62% sensitivity and with 68.6% sensitivity against *E. coli;* this in contrast with other studies which reported an overall 94% and 80% sensitivity [45,46]. Cefaclor has a spectrum of activity that resembles that of cephalexin with added activity against β-lactamase-producing *Haemophilus influenza* [47]. These suboptimal results may be due to the overuse of cefaclor in primary care settings for other infections such as pneumonia and upper respiratory tract infections, among others.

## 5. Conclusions

This study concluded that nitrofurantoin and ciprofloaxacin were suitable empirical treatments for uncomplicated urinary tract infections in the Jazan region of Saudi Arabia. The increased resistance of urinary tract pathogen isolates to TMPSMX and cefaclor was observed, implying that empirical therapy for the treatment of uUTIs with these agent should be reconsidered. Wide surveillance research is necessary to monitor effective empirical therapies and evaluate the regional antimicrobial susceptibility patterns.

Attempts should be considered to maximize therapeutic results; maintain cost-effective medical care; and minimize unwanted effects of antimicrobial use, such as toxicity, the selection of aggressive organisms, and the evolution of resistant bacterial strains.

## Figures and Tables

**Table 1:** Background sociodemographic characteristics of the included participants (120).

Background Characteristics		Frequency	%
Sex	Female	120	100%
Marital Status	Single	9	7.5
	Married	105	87.5
	Divorced	6	5.0
Risk factors	Type 2 diabetes mellitus	33	27.5
	Hypertension	12	10.0
	Renal stone	18	15
	Urinary catheter	6	5.0
	More than one risk factor	26	21.6

**Table 2:** Sensitivity test.

Drug	Frequency	%
Nitrofurantoin	110	91.7
Ciprofloaxcin	90	75.0
Trimethoprim/sulfamethoxazole	84	70.0
Cefaclor	75	62.5

**Table 3:** Antibiotic susceptibility patterns in relation to the causative organisms.

Drug		Microorganisms				
		*E. coli*	*Klebsiella pneumoniae*	*Staphylococcus* *aureus*	*Enterobacter* spp.	*Enterococcus* spp.
Total		105	6	3	3	3
Nitrofurantoin	Sensitivity	97 (92.4)	5 (83.3)	3 (100.0)	2 (66.7)	3 (100.0)
	Resistance	8 (7.6)	1 (16.7)	0 (0.0)	1 (33.3)	0 (0.0)
	*p* value	0.454	0.449	0.597	0.113	0.597
Ciprofloxacin	Sensitivity	75 (71.4)	6 (100.0)	3 (100.0)	3 (100.0)	3 (100.0)
	Resistance	30 (28.6)	0 (0.0)	0 (0.0)	0 (0.0)	0 (0.0)
	*p* value	0.017	0.147	0.311	0.311	0.311
Trimethoprim/sulfamethoxazole	Sensitivity	72 (68.6)	3 (50.0)	3 (100.0)	3 (100.0)	3 (100.0)
	Resistance	33 (31.4)	3 (50.0)	0 (0.0)	0 (0.0)	0 (0.0)
	*p* value	0.366	0.273	0.251	0.251	0.251
Cefaclor)	Sensitivity	72 (68.6)	0 (0.0)	0 (0.0)	0 (0.0)	3 (100.0)
	Resistance	33 (31.4)	6 (100.0)	3 (100.0)	3 (100.0)	0 (0.0)
	*p* value	0.000	0.001	0.024	0.024	0.174

**Table 4:** Correlation between antimicrobials and microorganisms using linear regression.

	Coefficient	*p* Value	95% CI
**Nitrofurantoin: R Square: 0.030**			
*E. coli*	0.924	0.000	0.870–0.978
*Klebsiella pneumoniae*	−0.090	0.440	−0.322–0.141
*Staphylococcus aureus*	0.076	0.641	−0.246–0.399
*Enterobacter* spp.	−0.257	0.117	−0.580–0.065
*Enterococcus* spp.	0.076	0.641	−0.246–0.399
**Ciprofloxacin: R Square: 0.048**			
*E. Coli*	0.714	0.000	0.631–0.798
*Klebsiella pneumoniae*	0.286	0.118	−0.073–0.645
*Staphylococcus aureus*	0.286	0.261	−0.215–0.786
*Enterobacter* spp.	0.286	0.261	−0.215–0.786
*Enterococcus* spp.	0.286	0.261	−0.215–0.786
**Trimethoprim/sulfamethoxazole: R Square: 0.043**			
*E. Coli*	0.686	0.000	0.597–0.774
*Klebsiella pneumoniae*	−0.186	0.336	−0.567–0.195
*Staphylococcus aureus*	0.314	0.244	−0.217–0.846
*Enterobacter* spp.	0.314	0.244	−0.217–0.846
*Enterococcus* spp.	0.314	0.244	−0.217–0.846
**Cefaclor: R Square: 0.195**			
*E. Coli*	0.686	0.000	0.600–0.771
*Klebsiella pneumoniae*	−0.686	0.000	−1.055–−0.317
*Staphylococcus aureus*	−0.686	0.009	−1.200–−0.171
*Enterobacter* spp.	−0.686	0.009	−1.200–−0.171
*Enterococcus* spp.	0.314	0.229	−0.200–0.829

## Data Availability

The data presented in this study are available on request from the corresponding author.
